# Pterostilbene attenuates osteoarthritis progression through p53-dependent autophagy activation: evidence from network analysis and experimental validation

**DOI:** 10.3389/fphar.2026.1686555

**Published:** 2026-01-23

**Authors:** Jiangping Wu, Chunpan Zhang, Yuanyuan Qin, Lixuan Zhu, Lingyan Liu, Fei Xu

**Affiliations:** 1 Department of Pain Management, The First People’s Hospital of Yunnan Province, Kunming, Yunnan, China; 2 The Affiliated Hospital of Kunming University of Science and Technology, Kunming, Yunnan, China; 3 Department of Obstetrics, The First People’s Hospital of Yunnan Province, Kunming, Yunnan, China

**Keywords:** autophagy, chondroprotection, network analysis, osteoarthritis, p53/AMPK/mTOR axis, pterostilbene

## Abstract

**Background:**

Osteoarthritis (OA) is a prevalent degenerative joint disease characterized by cartilage destruction and functional impairment. Dysregulated autophagy plays a pivotal role in chondrocyte apoptosis and extracellular matrix (ECM) degradation. Pterostilbene (PT), a natural polyphenolic metabolite with high bioavailability, exhibits potent anti-inflammatory and antioxidant activities. However, its precise role in regulating autophagy during OA progression remains unclear.

**Methods:**

Network analysis was employed to predict PT’s potential molecular targets and signaling pathways. To experimentally evaluate the drug-target interaction, the cellular thermal shift assay (CETSA) was performed. Functional validation was subsequently conducted *in vitro* using IL-1β-stimulated C28/I2 chondrocytes and *in vivo* in a monosodium iodoacetate-induced OA rat model. The p53 inhibitor pifithrin-α was applied to verify the mechanistic dependency.

**Results:**

Network analysis and molecular docking suggested p53 as a core target of PT. CETSA results supported the cellular target engagement of p53 by PT, showing that PT enhanced the thermal stability of p53 protein. In chondrocytes, PT mitigated IL-1β-induced ECM imbalance and apoptosis while enhancing Beclin1 expression and the LC3II/I ratio with reduced p62 accumulation. Mechanistically, PT promoted p53 nuclear accumulation, activated AMPK, and inhibited mTOR phosphorylation; these effects were attenuated by 3-methyladenine or pifithrin-α. *In vivo*, PT exhibited a dose-dependent chondroprotective effect, significantly reducing OARSI scores and restoring autophagy marker expression in cartilage tissue.

**Conclusion:**

This study demonstrates that PT exerts chondroprotective effects by activating autophagy through the p53/AMPK/mTOR axis, supported by evidence of specific p53 target engagement. These findings unveil a previously unrecognized molecular mechanism and underscore the translational potential of PT as a promising disease-modifying metabolite for OA therapy.

## 1 Introduction

Osteoarthritis (OA) is one of the most prevalent degenerative joint diseases worldwide, characterized by progressive cartilage erosion, joint deformity, chronic pain, and substantial loss of mobility that profoundly impair quality of life (Abramoff and Caldera, 2020; Jiang, 2022). The pathological hallmarks of OA include cartilage matrix degradation, reduced chondrocyte viability, and extracellular matrix (ECM) disorganization (van den Bosch, 2021; Wang et al., 2020; Kuwahara et al., 2022). Current clinical management primarily depends on nonsteroidal anti-inflammatory drugs (NSAIDs), intra-articular injections, and joint arthroplasty for advanced cases. However, these therapeutic approaches are palliative rather than curative, often accompanied by significant adverse effects, economic burden, and an inability to halt or reverse disease progression (Tuncay Duruöz et al., 2023; Fillingham et al., 2020).

Accumulating evidence indicates that dysregulated autophagy plays a central role in OA pathogenesis (Roelofs and De Bari, 2024; Kao et al., 2022). Cartilage tissues from OA patients display markedly suppressed chondrocyte autophagy, leading to the accumulation of dysfunctional mitochondria and misfolded proteins (Dalmao-Fernández et al., 2024; Duan et al., 2020). Such defects exacerbate oxidative and endoplasmic reticulum stress, thereby promoting chondrocyte apoptosis and accelerating structural cartilage deterioration (Loeser et al., 2016; Lu et al., 2024; Han et al., 2022). These findings collectively suggest that restoring autophagic flux may represent a promising strategy to mitigate chondrocyte apoptosis, enhance cartilage matrix anabolism, and ultimately slow OA progression (Xu et al., 2021; Liu et al., 2023).

The regulation of autophagy is primarily orchestrated through the AMPK/mTOR signaling cascade (Huang et al., 2023; Zhao et al., 2024), with the transcription factor p53 recognized as a pivotal upstream regulator (Rahman et al., 2022). Under conditions of cellular stress, p53 modulates autophagic flux by transcriptionally activating target genes such as Sestrin2, thereby governing the AMPK/mTOR axis (Chen et al., 2019). Notably, both the expression level and subcellular localization of p53 critically determine its functional outcomes, a regulatory mechanism that may play a fundamental role in the pathogenesis and progression of OA (Maiuri et al., 2010).

Natural products have gained increasing recognition for its therapeutic efficacy in OA management, with notable progress in the identification and pharmacological characterization of bioactive monomeric metabolites (Zhou et al., 2023). Pterostilbene (PT), a naturally occurring polyphenolic metabolite primarily found in blueberries and grapes, is a dimethylated analog of resveratrol distinguished by superior membrane permeability and bioavailability (Kim et al., 2020; Ko et al., 2015). PT exerts potent anti-inflammatory and antioxidant activities across multiple tissue types (Chen et al., 2024; He et al., 2018) and has been shown to induce antiproliferative responses in diverse malignancies via p53-dependent pathways (Wang and Attardi, 2022). In prostate carcinoma cells, PT activates AMPK signaling and orchestrates cell-cycle arrest and apoptotic cascades in a manner contingent on cellular p53 status (Lin et al., 2012).

Recent investigations have further revealed that PT attenuates inflammatory responses and suppresses reactive oxygen species (ROS) generation in chondrocytes (Xue et al., 2017; Lee et al., 2024), thereby highlighting its therapeutic potential in OA. However, the mechanistic basis through which PT modulates autophagy in OA—particularly via regulation of p53 expression and nucleocytoplasmic distribution—remains to be elucidated. Our network analysis-based preliminary findings suggest that PT may simultaneously engage autophagy-related and p53-associated signaling pathways, thereby providing a rational mechanistic foundation for subsequent experimental validation.

Based on the aforementioned evidence, PT is hypothesized to modulate autophagic activity through the p53/AMPK/mTOR signaling axis, thereby exerting potent chondroprotective effects in OA. To rigorously evaluate this hypothesis, an integrative research strategy combining network-based analysis, *in vitro* chondrocyte assays, and *in vivo* validation using a rat model of OA was adopted. Through this multilayered experimental framework, both mechanistic insight and empirical evidence supporting PT as a disease-modifying metabolite with translational potential for OA therapy were established.

## 2 Materials and methods

### 2.1 Network analysis

The Simplified Molecular Input Line Entry System (SMILES) format of PT, COC1 = CC(=CC(=C1)C=CC2 = CC = C(C=C2)O)OC, was retrieved from the PubChem database (https://pubchem.ncbi.nlm.nih.gov/) and submitted to the SwissADME platform (https://www.swissadme.ch/) to evaluate its physicochemical and drug-likeness profiles. The assessment included lipophilicity (consensus Log P_o_/*w* between −0.7 and +5.0), molecular size (molecular weight between 150 and 500 g/mol), polarity (TPSA between 20 and 130 Å^2^), aqueous solubility (log S ≤ −6), saturation (fraction of sp^3^-hybridized carbons ≥0.25), and molecular flexibility (≤9 rotatable bonds), in accordance with Lipinski’s rule-of-five criteria for rapid evaluation of drug-likeness.

OA-related genes were systematically collected from five disease-associated databases: GeneCards (https://www.genecards.org/, Relevance Score >1), Online Mendelian Inheritance in Man (OMIM, https://www.omim.org/), Therapeutic Target Database (TTD, https://db.idrblab.net/ttd/), DisGeNET (https://www.disgenet.org/), and Comparative Toxicogenomics Database (CTD, https://ctdbase.org/, Inference Score >20), using the keyword “osteoarthritis” for target retrieval across all databases. Potential molecular targets of PT were predicted using four established target-identification resources, namely, SwissTargetPrediction (https://swisstargetprediction.ch/), SuperPred (https://prediction.charite.de/), PharmMapper (https://lilab-ecust.cn/pharmmapper/), and CTD (https://ctdbase.org/), by searching with the term “pterostilbene.”

The predicted molecular targets of PT were intersected with OA-associated genesAfter removing duplicates, the common targets were identified and visualized using a Venn diagram generated with Venny 2.1 (https://bioinfogp.cnb.csic.es/tools/venny/). Subsequently, a phenotype–genotype correlation analysis was performed using VarElect (https://varelect.genecards.org/) to identify genes directly associated with OA, which were selected for downstream analyses as potential anti-OA targets.

The filtered intersection targets were uploaded to the DAVID 6.8 platform (https://david.ncifcrf.gov/) for Gene Ontology (GO) and Kyoto Encyclopedia of Genes and Genomes (KEGG) enrichment analyses, with *P* < 0.05 considered statistically significant. The enrichment results were visualized as bubble and bar plots using the Bioinformatics online tool (http://www.bioinformatics.com.cn/). GO analysis classified gene functions into three categories: biological processes (BP), cellular components (CC), and molecular functions (MF), whereas KEGG analysis identified key signaling pathways potentially associated with the pharmacological effects of PT in OA.

A protein-protein interaction (PPI) network was constructed using the STRING database (https://string-db.org/, confidence score ≥0.4) and visualized in Cytoscape (version 3.10.0). Key functional modules were identified using the MCODE plugin (Degree Cutoff = 2, Node Score Cutoff = 0.2, K-Core = 2), and hub proteins were screened using the cytoHubba plugin based on three topological parameters: betweenness, closeness, and degree (Yang et al., 2024). The top 10 ranked proteins from each algorithm were intersected to obtain the final hub targets, with unconnected nodes excluded from the final network.

The tissue distribution and expression patterns of core proteins were analyzed using the Human Protein Atlas (https://www.proteinatlas.org/), and the associations between key targets and autophagy were explored through VarElect and related network tools (https://varelect.genecards.org/, correlation score >5) (Cheng et al., 2022). The crystal structures of hub proteins (PDB IDs listed in Supplementary Table S1; resolution <3.0 Å) were retrieved from the RCSB Protein Data Bank (https://www.rcsb.org/). Molecular docking preprocessing, including water molecule removal, separation of co-crystallized ligands, addition of polar hydrogens, and generation of docking grid files, was performed using AutoDock Tools (version 1.5.6), which served as a preparatory interface for generating docking input files. Docking calculations were carried out using AutoDock Vina (version 1.2.2) under blind docking mode, where the grid box was expanded to encompass the entire protein surface, allowing unbiased exploration of potential binding sites. The docking complexes were visualized and analyzed using PyMOL (version 2.4). The docking workflow was designed solely as a predictive structural approach to illustrate potential PT-protein interactions, rather than as a quantitative evaluation of binding affinity.

### 2.2 Cell culture

The human chondrocyte cell line C28/I2 (CSI347Hu11, Cloud-Clone Corp., Wuhan, China) was cultured in DMEM/F12 medium supplemented with 10% FBS and 1% penicillin–streptomycin (100 U/mL) at 37 °C in a humidified incubator containing 5% CO_2_. Cells were seeded at a density of 2 × 10^5^ cells/mL in culture flasks, and the medium was renewed every 2–3 days. When cell confluence reached approximately 70%–80%, cells were detached using 0.25% trypsin-EDTA and passaged under standard aseptic conditions. Cells from passages 3–8 were used for all subsequent experiments.

### 2.3 Cell treatments and experimental groups

C28/I2 cells were treated with PT (HY-N0828, Lot# 130513, MedChemExpress, Shanghai, China) without further purification. The metabolite exhibited a purity of ≥99.90%, as verified by liquid chromatography–mass spectrometry (LC-MS). Its identity and purity were further confirmed based on structural consistency and analytical quality. Supporting data are provided in Supplementary Figures S1, S2. In cell viability assays, cells were exposed to PT at concentrations of 0, 5, 10, 20, 40, and 100 μM, with or without interleukin-1β (IL-1β, 10 ng/mL, HY-P7028, MedChemExpress, Shanghai, China), for 24 and 48 h. To assess PT’s effects on ECM metabolism, cells were treated with IL-1β (10 ng/mL) alone or in combination with PT (10, 20, or 40 μM) for 24 h.

To investigate the role of autophagy in PT-mediated chondroprotection, cells were divided into five groups: control (vehicle), IL-1β (10 ng/mL), IL-1β + PT (20 μM), IL-1β + rapamycin (RAPA, 100 nM; HY-10219, MedChemExpress, Shanghai, China), and IL-1β + PT + 3 MA (3-methyladenine, 5 mM; HY-19312, MedChemExpress, Shanghai, China). For mechanistic studies involving p53 transcriptional activity, pifithrin-α (PFT-α, 20 μM; HY-15484, MedChemExpress, Shanghai, China), a selective inhibitor of p53-dependent transcription, was added 2 h prior to other treatments. All treatments were performed for 24 h unless otherwise specified. PT and other compounds were dissolved in DMSO, and the final DMSO concentration was maintained below 0.1% in all experimental groups. Control cells received an equivalent volume of vehicle.

### 2.4 Cell viability assay

Cell viability was determined using the CCK-8 assay kit (CK04, Dojindo, Kumamoto, Japan). Cells were seeded in 96-well plates at a density of 2 × 10^3^ cells/well and cultured for 24 h to allow complete attachment. Subsequently, cells were treated with PT alone or co-treated with IL-1β and PT for 24 or 48 h. After treatment, 10 μL of CCK-8 solution was added to each well and incubated at 37 °C for 1 h in the dark. The optical density (OD) was measured at 450 nm with a reference wavelength of 630 nm using a microplate reader (Bio-Rad iMark, Hercules, CA, United States). Relative cell viability was calculated as the percentage of control group OD values, and each experiment was performed independently in triplicate.

### 2.5 Western blotting (WB)

Nuclear and cytoplasmic proteins were extracted using a nuclear extraction kit (78835, Thermo Fisher Scientific, Waltham, MA, United States). Cells were incubated on ice with cytoplasmic extraction buffer for 10 min, followed by centrifugation, and the resulting nuclear pellets were sonicated in nuclear extraction buffer prior to a second centrifugation. For total protein extraction, cells were lysed in RIPA buffer supplemented with a protease inhibitor cocktail (1:100, HY-K0010, MedChemExpress, Shanghai, China) and phosphatase inhibitors (1:50, HY-K0021, MedChemExpress, Shanghai, China) for 30 min on ice, and then centrifuged at 12,000 × g for 15 min at 4 °C. Cartilage tissues from rat knees (8 weeks post-induction) were pulverized in liquid nitrogen, homogenized in RIPA buffer containing the same inhibitors, lysed for 2 h at 4 °C, and centrifuged at 14,000 × g for 20 min at 4 °C. Protein concentrations were measured using the BCA Protein Assay Kit (PC0020, Solarbio, Beijing, China).

Equal amounts of protein (30 μg) were separated by 8%–12% SDS–PAGE and transferred onto PVDF membranes. Membranes were blocked with 5% bovine serum albumin (BSA) in TBST for 1 h and incubated overnight at 4 °C with the following primary antibodies: LC3B (cells 1:1000, tissue 1:800; #3868, Cell Signaling Technology(CST), Danvers, MA, United States), Beclin1 (cells 1:1000, tissue 1:800; #3495, CST), p62 (cells 1:1000, tissue 1:800; #88588, CST), collagen type II alpha 1 chain (COL2A1; cells 1:1000, tissue 1:500; #ab34712, Abcam, Cambridge, UK), aggrecan (ACAN; cells 1:500, tissue 1:500; #sc-33695, Santa Cruz Biotechnology, Dallas, TX, United States), matrix metalloproteinase 3 (MMP3; cells 1:1000, tissue 1:500; #14351, CST), matrix metalloproteinase 13 (MMP13; cells 1:1000, tissue 1:500; #69926, CST), p-AMPKα (Thr172; 1:1000; #2535, CST), AMPKα (1:1000; #2532, CST), p-mTOR (Ser2448; 1:1000; #5536, CST), mTOR (1:1000; #2983, CST), p53 (1:1000; #2527, CST), PCNA (1:2000; #13110, CST), and GAPDH (cells 1:10,000, tissue 1:5000; #5174, CST). After washing with TBST, membranes were incubated with HRP-conjugated secondary antibodies (1:5000, ab6721, Abcam) for 1 h at room temperature. Protein bands were visualized using an enhanced chemiluminescence substrate (MilliporeSigma, Burlington, MA, United States) and imaged with a ChemiDoc MP Imaging System (Bio-Rad Laboratories, Hercules, CA, United States). Band intensities were quantified using ImageJ software (version 1.53a), with GAPDH as the internal loading control. All experiments were performed independently in triplicate.

### 2.6 Cell apoptosis detection

Apoptotic DNA fragmentation was evaluated using a TUNEL assay kit (MK1023, Dojindo, Kumamoto, Japan). Cells were seeded onto sterile glass coverslips placed in 24-well plates and subjected to the indicated treatments. After treatment, cells were fixed with 4% paraformaldehyde for 20 min at room temperature and permeabilized with 0.3% Triton X-100 on ice for 8 min. For positive controls, cells were treated with DNase I (3 U/mL) for 15 min at room temperature. The TUNEL reaction mixture was prepared following the manufacturer’s instructions and applied to the cells for 45 min at 37 °C in a humidified, light-protected chamber.

After washing with PBS, nuclei were counterstained with DAPI (1:5000) for 5 min. Fluorescent images from eight randomly selected fields per coverslip were captured using a fluorescence microscope (BX51, Olympus, Tokyo, Japan) equipped with a ×20 objective. TUNEL-positive cells (red fluorescence) and total nuclei (DAPI-stained, blue fluorescence) were counted, and the percentage of apoptotic cells was calculated accordingly. Each experiment was performed in triplicate wells and repeated independently three times.

### 2.7 p53 subcellular localization

Drug-treated cells were fixed with 4% paraformaldehyde for 15–30 min at room temperature, permeabilized with 0.3% Triton X-100 on ice for 5–10 min, and rinsed three times with PBS. After blocking with 5% bovine serum albumin (BSA) for 30 min, cells were incubated overnight at 4 °C with a primary antibody against p53 (1:500, #2527, CST, Danvers, MA, United States), followed by washing with PBS and incubation with an Alexa Fluor 594-conjugated secondary antibody (1:500, #A-11012, Invitrogen, Carlsbad, CA, United States) for 1 h in the dark at room temperature. After washing, nuclei were counterstained with DAPI (1 μg/mL) for 5–10 min, and samples were mounted using an anti-fade fluorescence mounting medium and imaged with a confocal microscope (T2i, Nikon, Tokyo, Japan). Images were captured using a ×40 oil-immersion objective, with excitation wavelengths of 405 nm for DAPI and 561 nm for Alexa Fluor 594, and emission spectra collected at 420–480 nm and 580–630 nm, respectively. The subcellular distribution of p53 between nuclear and cytoplasmic compartments was visually assessed.

### 2.8 Cellular thermal shift assay (CETSA)

To validate the direct physical interaction between PT and p53 within a cellular environment, a CETSA was performed as previously described. Briefly, C28/I2 chondrocytes were incubated with PT (20 μM) or vehicle (DMSO) for 6 h. Subsequently, cells were harvested, washed with PBS, and resuspended in PBS supplemented with protease inhibitors. The cell suspensions were aliquoted and subjected to a thermal gradient ranging from 38 °C to 70 °C (nine discrete temperature points) for 3 min, followed by immediate cooling at room temperature for 3 min. Cells were then lysed by three freeze-thaw cycles using liquid nitrogen. The lysates were centrifuged at 20,000 × g for 20 min at 4 °C to separate the soluble fractions, which were subsequently analyzed via Western blotting. The thermal stability of p53 was assessed by Western blotting, and protein band intensities were normalized to the values at 38 °C to generate thermal aggregation curves.

### 2.9 Animal experiment

Animal care and experimental procedures were performed in accordance with the National Institutes of Health Guide for the Care and Use of Laboratory Animals (2011). Eight-week-old male Sprague–Dawley (SD) rats (220 ± 20 g, specific pathogen-free grade; n = 72) were obtained from the Laboratory Animal Center of Kunming University of Science and Technology (Kunming, China) and randomly assigned to six groups (12 rats per group): sham control (Sham), MIA-induced OA model (MIA), MIA + low-dose PT (PT-L, 10 mg/kg), MIA + medium-dose PT (PT-M, 20 mg/kg), MIA + high-dose PT (PT-H, 40 mg/kg), and MIA + RAPA (3 mg/kg). All animal protocols were approved by the Animal Ethics Committee of Kunming University of Science and Technology.

The OA model was established by intra-articular injection of monosodium iodoacetate (MIA, S8251, Sigma-Aldrich, St. Louis, MO, United States), which primarily mimics early-stage OA through chondrocyte apoptosis and proteoglycan depletion. Rats were anesthetized with 1.5% isoflurane and positioned supine, the right knee region was shaved, disinfected, and 3 mg MIA dissolved in 50 μL sterile saline was injected into the joint cavity through the infrapatellar ligament using a 27G needle (Mao et al., 2021). The sham group received an equivalent volume of sterile saline following the same procedure.

Drug interventions commenced on day 3 after model induction and were administered every other day until sacrifice. PT dosages were based on previously reported effective concentrations in animal studies (Xue et al., 2017; Rui et al., 2022) and were adjusted to evaluate potential dose-dependent effects in the OA model. RAPA served as a positive control for autophagy activation (Bao et al., 2020; Tang et al., 2021). All drugs were administered intraperitoneally, with PT dissolved in DMSO and diluted in sterile saline to achieve a final DMSO concentration below 1%. Control animals (Sham and MIA groups) received equivalent volumes of vehicle solution. Eight weeks after surgery, all rats were euthanized under pentobarbital sodium anesthesia (150 mg/kg), and knee joint specimens were harvested and fixed in 4% paraformaldehyde for histological and molecular analyses.

### 2.10 Histology and immunohistochemistry

At week 8, rats were euthanized under pentobarbital sodium anesthesia (150 mg/kg). The right knee joints were harvested, fixed in 4% paraformaldehyde for 24 h, and decalcified in 12% EDTA for 6 weeks. Paraffin-embedded sections (5 μm thick) were prepared for hematoxylin-eosin (H&E) staining, Safranin O-Fast Green staining, and immunohistochemistry (IHC). For IHC, sections were incubated overnight at 4 °C with primary antibodies against p62 (1:200, #88588, CST, Danvers, MA, United States) and ACAN (1:200, #sc-33695, Santa Cruz Biotechnology, Dallas, TX, United States), followed by incubation with an HRP-conjugated secondary antibody (1:500, #GB23303, Servicebio, Wuhan, China) for 1 h at room temperature, and visualization using 3,3′-diaminobenzidine. Images were captured using a Leica microscope (DM2000, Leica Microsystems Wetzlar GmbH, Wetzlar, Germany), and positively stained cells were quantified using Image-Pro Plus software (version 6.0).

Cartilage degeneration was assessed using the Osteoarthritis Research Society International (OARSI) scoring system (24-point scale), calculated as the product of lesion severity (0–6) and extent (0–4) (Ye et al., 2024). Evaluation criteria included surface fissures, cellular organization, matrix staining, and subchondral bone integrity. Three sections per sample and five randomly selected fields (×200 magnification) per section were analyzed by two independent, blinded histopathologists. Data were expressed as the mean ± standard error of the mean (SEM).

### 2.11 Statistical analysis

Statistical analyses were performed using SPSS software (version 29.0). Data are expressed as the mean ± SEM. For normally distributed data, one-way analysis of variance (ANOVA) followed by appropriate *post hoc* comparisons was applied. For non-normally distributed data, the Kruskal–Wallis test followed by Dunn’s multiple-comparison test was used. Differences were considered statistically significant at *P* < 0.05. Each experiment was performed independently at least three times. All statistical graphs were generated using GraphPad Prism software (version 9.0).

## 3 Results

### 3.1 Network analysis

#### 3.1.1 Drug properties and target prediction of PT

**FIGURE 1:**
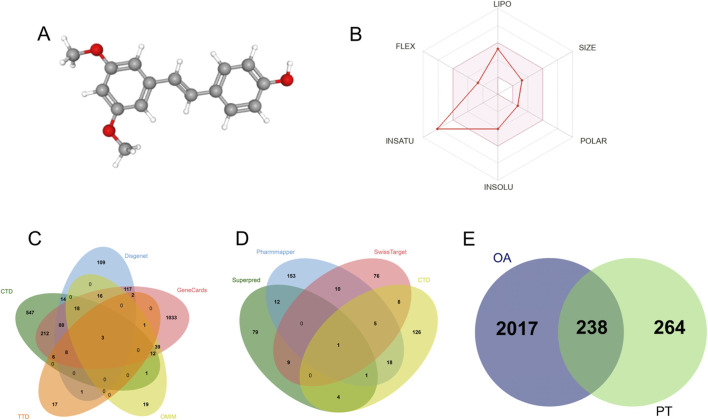
Drug-likeness assessment and target prediction of PT. **(A)** Three-dimensional molecular structure of PT; **(B)** Bioavailability radar plot showing drug-like features (LIPO, FLEX, SIZE, POLAR, INSOLU, INSATU); **(C)** Screening of OA-related genes from multiple databases (CTD, GeneCards, DisGeNET, OMIM, TTD); **(D)** Predicted PT targets obtained from PharmMapper, SwissTargetPrediction, SuperPred, and CTD; **(E)** Venn diagram illustrating overlapping genes between OA-related and PT-target datasets. Numbers in panels **(C–E)** indicate the counts of unique or overlapping genes.

The molecular structure of PT (Figure 1A) was retrieved from the PubChem database (CID 5281727). PT exhibits a typical stilbene backbone composed of two phenyl rings linked by an ethylenic bridge, with methoxy and hydroxyl substituents. The bioavailability radar (Figure 1B) provides a comprehensive visualization of PT’s physicochemical profile, indicating a well-balanced distribution across lipophilicity, polarity, molecular size, solubility, and flexibility parameters. Quantitative descriptors (Supplementary Figure S3) demonstrate that PT displays favorable physicochemical characteristics, including a molecular weight of 256.30 g/mol, a lipid–water partition coefficient (CLogP = 3.31), and a polar surface area (PSA = 38.69 Å^2^). PT complies with all major drug-likeness rules (Lipinski, Veber, Egan, Muegge, and Ghose). In silico pharmacokinetic evaluation further predicts high gastrointestinal absorption, positive blood–brain barrier permeability (BBB+), a bioavailability score of 0.55, and synthetic accessibility of 2.29, collectively supporting PT’s potential as a drug-like bioactive metabolite.

Through integrated network analysis, the putative molecular targets of PT in OA were systematically identified. A total of 2,255 OA-related genes were compiled from multiple disease–gene databases (Figure 1C). Meanwhile, 502 predicted PT targets were retrieved from target prediction platforms (Figure 1D). The intersection of these datasets yielded 238 overlapping targets, which may represent core mediators of PT’s therapeutic actions in OA and served as the foundation for subsequent enrichment and mechanistic analyses (Figure 1E).

#### 3.1.2 Functional enrichment and molecular interaction network analysis

**FIGURE 2:**
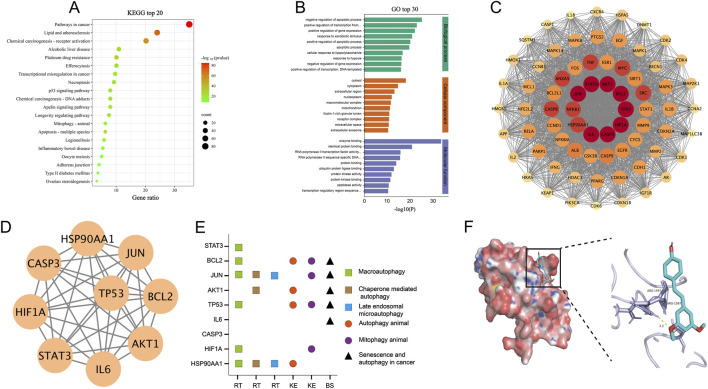
Network analysis of PT’s regulatory mechanisms in autophagy. **(A)** Top 20 KEGG-enriched pathways; **(B)** Top 30 GO enrichment terms across BP, CC, and MF categories; **(C)** PPI network highlighting key nodes in red, with node importance represented by color intensity; **(D)** Core regulatory network of hub targets; **(E)** Distribution of key targets across autophagy-related pathways derived from the Reactome (RT), KEGG (KE), and NCBI BioSystems (BS) databases; **(F)** Molecular docking analysis between PT and TP53 showing the overall protein surface (left) and a magnified view of the binding interface (right).

Functional enrichment analysis of the 238 overlapping targets revealed that, according to KEGG pathway analysis, these genes were predominantly enriched in pathways associated with cancer, the p53 signaling pathway, and autophagy-related pathways (Figures 2A,B). GO enrichment analysis further demonstrated that these targets were mainly involved in the regulation of apoptosis and transcriptional processes within the biological process category, were primarily localized to the extracellular region and cytoplasm under the cellular component category, and exhibited enrichment in enzyme binding and protein binding activities in the molecular function category.

To further elucidate the potential interactions among these targets, a PPI network was constructed (Figure 2C), and topological analysis integrating multiple centrality algorithms identified nine hub genes—AKT1, STAT3, TP53, IL6, JUN, HIF1A, IL1B, HSP90AA1, and CASP3—as key regulatory nodes. The interaction network among these core targets (Figure 2D) delineates a central signaling cluster that PT may modulate to exert its therapeutic effects in OA.

#### 3.1.3 Autophagy pathway association and molecular docking analysis

Further analysis of the association between key targets and autophagy pathways revealed that, except for CASP3, the remaining genes are extensively involved in multiple autophagy processes, including macroautophagy, chaperone-mediated autophagy, microautophagy, mitophagy, and senescence-associated autophagy (Figure 2E). The specific autophagy relevance scores for each target are summarized in Supplementary Table S1. Molecular docking analysis demonstrated that PT exhibited binding affinity toward all nine hub proteins (Supplementary Figure S4). TP53 ranked among the top predicted targets, exhibiting one of the lowest docking energy scores (−7.3 kcal/mol) (Figure 2F; Supplementary Table S2), suggesting a preferential molecular interaction between PT and TP53. These findings further support the hypothesis that PT may regulate autophagy through modulation of the p53 signaling pathway. Collectively, the network analysis and molecular docking results indicate that PT targets multiple autophagy-related pathways, particularly via TP53, thereby providing a theoretical foundation for subsequent mechanistic validation of PT in OA treatment.

### 3.2 Concentration-dependent effects of PT on chondrocyte viability and ECM metabolism

**FIGURE 3:**
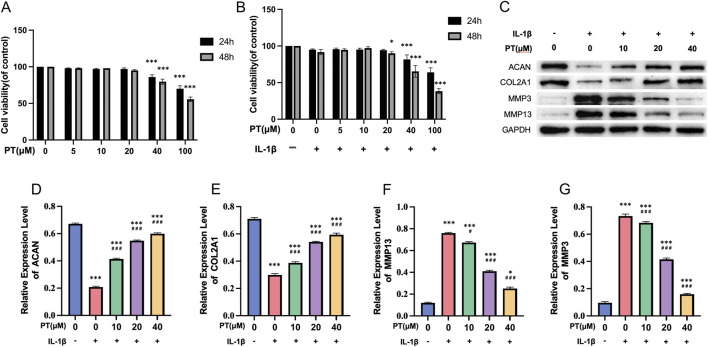
Effects of PT on chondrocyte viability and ECM metabolism under IL-1β stimulation. **(A,B)** Cell viability determined by CCK-8 assay in chondrocytes treated with increasing PT concentrations (0, 5, 10, 20, 40, 100 μM) for 24 and 48 h without **(A)** or with **(B)** IL-1β (10 ng/mL) stimulation; **(C)** Representative WB images showing ACAN, COL2A1, MMP3 and MMP13 expression; **(D–G)** Quantitative analysis of protein levels normalized to GAPDH for ACAN **(D)**, COL2A1 **(E)**, MMP13 **(F)**, and MMP3 **(G)**. Data = mean ± SEM (n = 3). *P < 0.05, ***P < 0.001 vs. control; #P < 0.05, ###P < 0.001 vs. IL-1β.

To determine the safe and effective concentration range of PT for OA treatment, the effects of PT on chondrocyte viability were evaluated (Figures 3A,B). Under normal culture conditions, PT at 5–20 μM showed no significant difference in cell survival compared with the control group (*P* > 0.05), whereas concentrations ≥40 μM markedly reduced viability at both 24 and 48 h (*P* < 0.001). Under IL-1β stimulation, PT at 10 μM had no effect on cell viability, 20 μM caused only a mild reduction at 48 h (*P* < 0.05), while 40 and 100 μM markedly inhibited cell survival (*P* < 0.001). These data indicate that PT exerts a clear concentration-dependent effect, highlighting the necessity of selecting an appropriate dosage to balance efficacy and cytotoxicity.

As shown in Figures 3C–G, IL-1β stimulation disrupted ECM homeostasis, markedly downregulating the cartilage matrix proteins ACAN and COL2A1 while upregulating the catabolic enzymes MMP3 and MMP13 (all *P* < 0.001 vs. control). PT treatment restored this imbalance in a concentration-dependent manner: as the concentration increased from 10 to 40 μM, ACAN and COL2A1 expression progressively recovered, whereas MMP3 and MMP13 levels declined. Even at 10 μM, PT significantly attenuated IL-1β-induced ECM degradation (*P* < 0.05), and the protective effect became more pronounced at 20 μM (*P* < 0.001). Although 40 μM produced the most robust restorative effect, it simultaneously compromised cell viability.

Considering both efficacy and cytotoxicity, PT at 20 μM exhibited the optimal therapeutic index, significantly alleviating IL-1β-induced ECM metabolic disturbance without markedly affecting chondrocyte viability. This concentration was therefore selected for subsequent mechanistic experiments. Collectively, these findings suggest that PT exerts anti-OA effects by preserving ECM metabolic balance in chondrocytes, thereby establishing a solid foundation for exploring its autophagy-regulatory mechanism.

### 3.3 PT protects chondrocytes through autophagy

**FIGURE 4:**
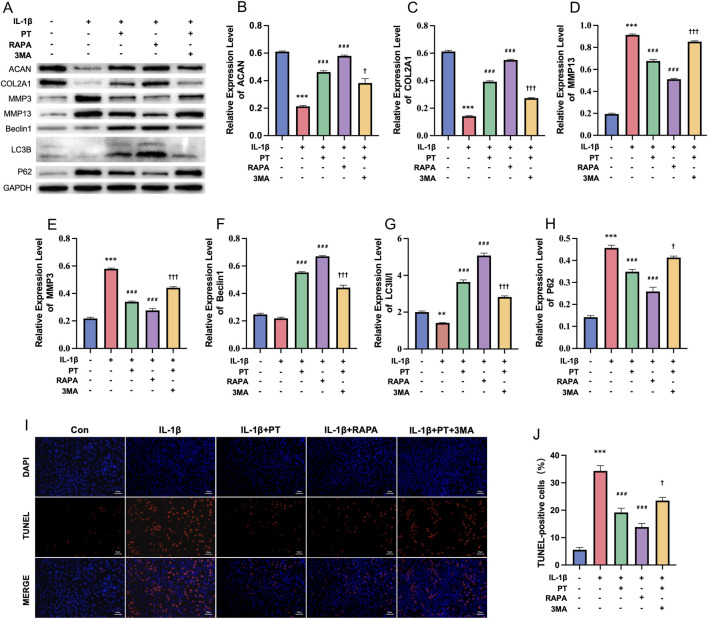
PT protects chondrocytes against IL-1β-induced damage via autophagy activation. **(A)** Representative WB images showing ACAN, COL2A1, MMP3, MMP13, Beclin1, LC3B, and p62 expression; **(B–H)** Quantitative analysis of protein levels normalized to GAPDH for ACAN **(B)**, COL2A1 **(C)**, MMP13 **(D)**, MMP3 **(E)**, Beclin1 **(F)**, LC3II/I ratio **(G)**, and p62 **(H)**; **(I)** Representative TUNEL staining showing apoptotic cells (red) and nuclei (blue) (scale bar = 50 μm); **(J)** Percentage of TUNEL-positive cells. Data = mean ± SEM (n = 3). **P < 0.01, ***P < 0.001 vs. control; ###P < 0.001 vs. IL-1β; †P < 0.05, †††P < 0.001 vs. IL-1β + PT.

To examine whether PT protects chondrocytes through autophagy activation, the expression of cartilage matrix proteins, matrix-degrading enzymes, and autophagy-related markers was evaluated under different treatment conditions (Figure 4A). WB analysis revealed that, compared with the control group, IL-1β markedly decreased ACAN and COL2A1 expression while upregulating MMP3 and MMP13 levels (all *P* < 0.001). PT treatment effectively counteracted these alterations (all *P* < 0.001 vs. IL-1β), exhibiting effects comparable to the autophagy activator RAPA. Notably, co-treatment with the autophagy inhibitor 3 MA partially reversed PT’s protective effects, resulting in a mild decrease in ACAN (*P* < 0.05), a significant reduction in COL2A1 (*P* < 0.001), and significant increases in MMP3 and MMP13 (*P* < 0.001) (Figures 4B–E).

At the same time, IL-1β treatment decreased the LC3II/I ratio *(P* < 0.01) and elevated p62 expression (*P* < 0.001), with no significant change in Beclin1 levels. In contrast, PT treatment significantly upregulated Beclin1 expression and LC3II/I ratio while reducing p62 levels (all *P* < 0.001), indicating autophagy activation. 3MA co-treatment markedly inhibited these pro-autophagic effects of PT, as evidenced by decreased Beclin1 expression and LC3II/I ratio (both *P* < 0.001) and increased p62 levels (*P* < 0.05) (Figures 4F–H).

To further verify the involvement of autophagy in PT-mediated cytoprotection, TUNEL staining was performed. As shown in Figures 4I,J, IL-1β markedly increased the proportion of TUNEL-positive cells (34.5% ± 2.3% vs. 5.2% ± 1.1% in control, *P* < 0.001). Both PT and RAPA treatments significantly reduced IL-1β-induced apoptosis (18.5% ± 1.4% and 13.8% ± 1.2%, respectively; *P* < 0.001), whereas 3 MA co-treatment partially abrogated PT’s anti-apoptotic effect (23.4% ± 1.6%, *P* < 0.05). Collectively, these findings demonstrate that PT protects chondrocytes from IL-1β-induced injury and apoptosis primarily through activation of the autophagy pathway.

### 3.4 PT activates autophagy via the p53/AMPK/mTOR signaling axis

**FIGURE 5:**
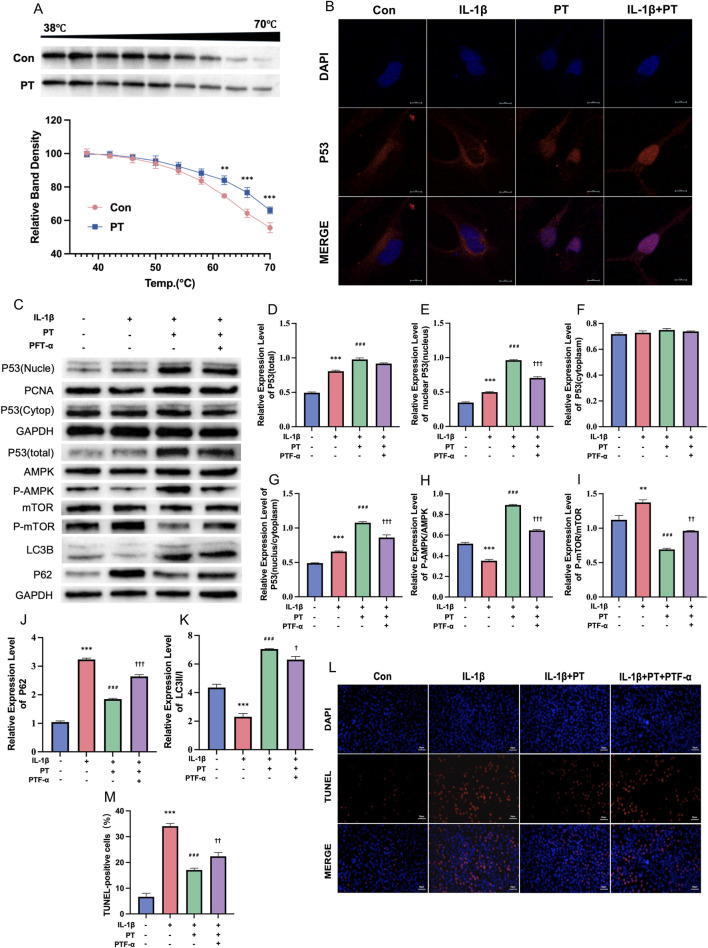
PT modulates p53 localization, AMPK/mTOR signaling, and autophagy-related markers in chondrocytes. **(A)** CETSA results assessing the thermal stability of p53 in C28/I2 cells treated with PT or vehicle across a temperature range of 38 °C–70 °C. Representative WB bands are shown above the normalized thermal aggregation curves; **(B)** Representative immunofluorescence confocal microscopy images illustrating p53 subcellular localization (red) and DAPI-stained nuclei (blue) in chondrocytes across different treatment groups (scale bar = 10 μm); **(C)** Representative WB bands showing p53 distribution (nuclear, cytoplasmic, and total), the phosphorylation status of AMPK and mTOR, and autophagy markers (LC3 and p62); **(D–K)** Quantitative analyses of protein expression levels: total p53/GAPDH **(D)**, nuclear p53/PCNA **(E)**, cytoplasmic p53/GAPDH **(F)**, nuclear/cytoplasmic p53 ratio **(G)**, p-AMPK/AMPK **(H)**, p-mTOR/mTOR **(I)**, p62/GAPDH **(J)**, and the LC3II/I ratio **(K)**; **(L)** Representative TUNEL staining images showing apoptotic cells (red) and nuclei (blue) (scale bar = 50 μm); **(M)** Quantitative analysis of the percentage of TUNEL-positive cells across groups. Data = mean ± SEM (n = 3). **P < 0.01, ***P < 0.001 vs. control; ###P < 0.001 vs. IL-1β; †P < 0.05, ††P < 0.01, †††P < 0.001 vs. IL-1β + PT.

To elucidate the molecular mechanism underlying PT-mediated chondroprotection, a CETSA was performed to evaluate whether PT engages p53 in C28/I2 chondrocytes. As shown in Figure 5A, p53 protein in the vehicle-treated group exhibited a rapid temperature-dependent decrease, indicative of thermal denaturation. In contrast, PT treatment markedly enhanced the thermal stability of p53, resulting in a significantly greater preservation of the soluble protein fraction at elevated temperatures (62 °C–70 °C). Quantitative analysis demonstrated a significant rightward shift of the p53 thermal aggregation curve in the presence of PT (P < 0.01), supporting PT-induced cellular target engagement with p53.

To further determine whether PT-induced p53 engagement affects its subcellular distribution, immunofluorescence staining and subcellular fractionation were performed. Confocal microscopy (Figure 5B) revealed that PT markedly promoted p53 nuclear accumulation under both basal and IL-1β-stimulated conditions, whereas p53 localization remained predominantly cytoplasmic in control and IL-1β-treated cells. Consistently, Western blot analysis of nuclear and cytoplasmic fractions (Figure 5C) showed that IL-1β modestly increased total and nuclear p53 levels without significantly affecting cytoplasmic p53, resulting in a moderate elevation of the nuclear-to-cytoplasmic ratio (Figures 5D–G, *P* < 0.001). PT treatment further enhanced nuclear p53 accumulation and total p53 expression, leading to a pronounced increase in the nuclear-to-cytoplasmic ratio (*P* < 0.001). Notably, co-treatment with the p53 transcriptional inhibitor PFT-α significantly attenuated PT-induced nuclear p53 accumulation and reduced the nuclear-to-cytoplasmic ratio (*P* < 0.001).

Concurrently, IL-1β stimulation suppressed AMPK phosphorylation and increased mTOR phosphorylation (Figures 5C,H,I). PT treatment significantly reversed these alterations by enhancing AMPK phosphorylation and inhibiting mTOR activation (*P* < 0.001). Importantly, PFT-α co-treatment partially abrogated PT-mediated modulation of the AMPK/mTOR signaling pathway, indicating that p53 nuclear accumulation and transcription-dependent signaling are required for PT-induced AMPK activation and mTOR inhibition.

Consistent with these signaling changes, IL-1β exposure markedly increased p62 accumulation and reduced the LC3II/I ratio (Figures 5C,J,K, *P* < 0.001). PT treatment effectively counteracted these effects, as evidenced by decreased p62 levels and an elevated LC3II/I ratio (*P* < 0.001). These autophagy-related effects were partially reversed by PFT-α co-treatment, resulting in increased p62 expression (*P* < 0.001) and a reduced LC3II/I ratio (*P* < 0.05). Furthermore, TUNEL staining (Figures 5L,M) demonstrated that IL-1β significantly increased chondrocyte apoptosis compared with control cells (33.6% ± 1.3% vs. 6.5% ± 1.4%, *P* < 0.001), whereas PT treatment markedly reduced apoptotic cell numbers (16.2% ± 0.9%, *P* < 0.001). This cytoprotective effect was partially diminished by PFT-α co-treatment (21.8% ± 1.4%, *P* < 0.01). Collectively, these findings demonstrate that PT engages and stabilizes p53, promotes its nuclear accumulation, and activates a p53-dependent AMPK/mTOR signaling cascade, thereby enhancing autophagy-related responses and suppressing apoptosis in chondrocytes.

### 3.5 PT ameliorates OA in a dose-dependent manner by enhancing autophagy

**FIGURE 6:**
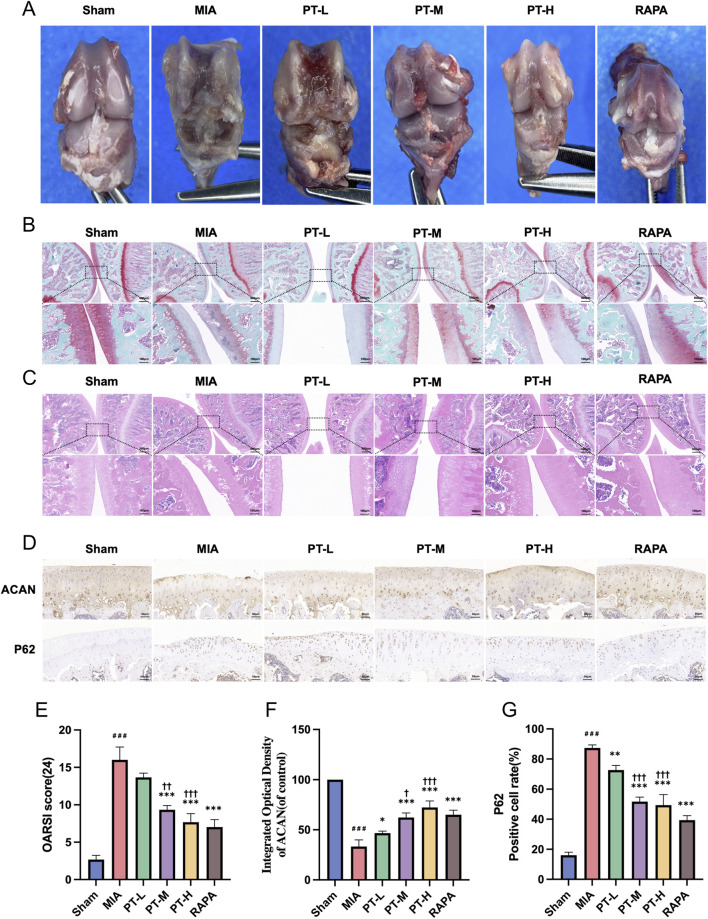
PT ameliorates MIA-induced OA through dose-dependent autophagy activation *in vivo*. **(A)** Macroscopic knee-joint appearance in all treatment groups; **(B,C)** Safranin O/Fast Green and H&E staining showing dose-dependent structural alterations (scale bars: upper = 500 μm, lower = 100 μm); **(D)** IHC staining for ACAN and p62 (scale bar = 50 μm); **(E)** OARSI scores quantifying cartilage damage; **(F)** ACAN immunostaining intensity; **(G)** Percentage of p62-positive chondrocytes. Data = mean ± SEM (n = 3). ###P < 0.001 vs. sham; *P < 0.05, **P < 0.01, ***P < 0.001 vs. MIA; †P < 0.05, ††P < 0.01, †††P < 0.001 vs. PT-L.

To verify the dose-dependent protective effects of PT *in vivo*, an MIA-induced early-stage OA rat model was established and treated with graded PT dosing regimens. As shown in Figure 6A, MIA injection resulted in pronounced cartilage erosion characterized by roughened joint surfaces and structural degradation. PT administration ameliorated these pathological changes in a dose-dependent manner: PT-L (10 mg/kg) provided only modest surface protection with visible irregularities, PT-M (20 mg/kg) produced moderate improvement with smoother surfaces, and PT-H (40 mg/kg) yielded marked restoration approaching normal joint morphology. RAPA treatment produced comparable joint surface preservation to that observed in the high-dose PT group.

Histological evaluation using Safranin O-Fast Green (Figure 6B) and H&E staining (Figure 6C) revealed that the MIA group exhibited severely thinned cartilage with irregular architecture and depleted proteoglycan content. Cartilage preservation became apparent at the medium dose (PT-M), which showed notable recovery in thickness and proteoglycan intensity compared with PT-L. PT-H achieved substantial histological improvement with near-normal cartilage thickness and cellular organization, while RAPA treatment exhibited a similar degree of protection. Quantitative assessment using the OARSI scoring system further confirmed the dose-dependent chondroprotective effects of PT (Figure 6E). The MIA group displayed significantly higher OARSI scores than the sham group (16.0 ± 1.7 vs. 2.4 ± 0.4, *P* < 0.001). PT-L (13.7 ± 0.6) showed no significant improvement relative to MIA, suggesting that 10 mg/kg represents a subtherapeutic dose. In contrast, PT-M (9.3 ± 0.6, *P* < 0.001 vs. MIA, *P* < 0.01 vs. PT-L) and PT-H (7.7 ± 1.2, *P* < 0.001 vs. MIA, *P* < 0.001 vs. PT-L) provided progressively enhanced protection, while RAPA achieved the most pronounced therapeutic efficacy (7.0 ± 1.0, *P* < 0.001 vs. MIA).

IHC analysis (Figures 6D,F,G) revealed that MIA induction significantly decreased ACAN expression to 33.3% ± 6.7% of control levels (*P* < 0.001) and increased p62-positive cells to 87.3% ± 2.1% (*P* < 0.001), indicating cartilage matrix degradation and autophagy inhibition. PT treatment restored these markers in a dose-dependent manner: PT-L (10 mg/kg) modestly increased ACAN to 46.7% ± 2.1% (*P* < 0.05 vs. MIA) and reduced p62-positive cells to 72.7% ± 3.1% (*P* < 0.01 vs. MIA); PT-M (20 mg/kg) produced significant improvements, restoring ACAN to 62.3% ± 4.5% (*P* < 0.001 vs. MIA, *P* < 0.05 vs. PT-L) and lowering p62-positive cells to 51.7% ± 3.1% (*P* < 0.001 vs. MIA, *P* < 0.001 vs. PT-L); PT-H (40 mg/kg) provided further gains, elevating ACAN to 72.3% ± 6.5% (*P* < 0.001 vs. MIA, *P* < 0.001 vs. PT-L) and reducing p62-positive cells to 49.3% ± 7.0% (*P* < 0.001 vs. MIA, *P* < 0.001 vs. PT-L). RAPA achieved comparable molecular restoration (ACAN 65.0% ± 4.6%; p62-positive cells 39.3% ± 3.1%; both *P* < 0.001 vs. MIA). These findings demonstrated dose-dependent chondroprotection via autophagy activation and delineated a therapeutic window of 20–40 mg/kg for optimal OA intervention.

**FIGURE 7:**
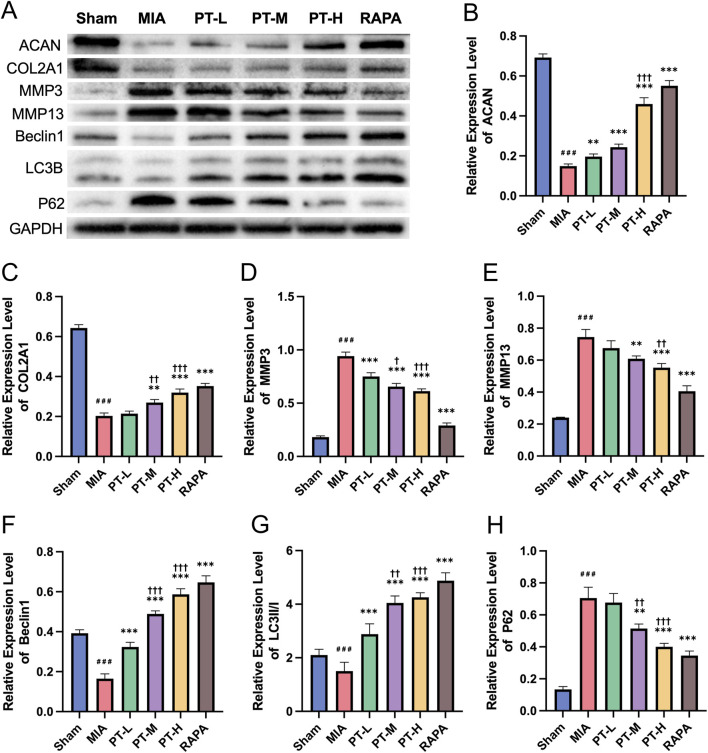
PT regulates cartilage-specific and autophagy-related protein expression in MIA-induced OA *in vivo*. **(A)** Representative WB images showing ACAN, COL2A1, MMP3, MMP13, Beclin1, LC3B, and p62 expression; **(B–H)** Quantitative analysis of protein levels normalized to GAPDH for ACAN **(B)**, COL2A1 **(C)**, MMP3 **(D)**, MMP13 **(E)**, Beclin1 **(F)**, LC3II/I ratio **(G)**, and p62 **(H)**. Data = mean ± SEM (n = 3). ###P < 0.001 vs. sham; *P < 0.01, **P < 0.001 vs. MIA; †P < 0.05, ††P < 0.01, †††P < 0.001 vs. PT-L.

*In vivo* WB analysis (Figure 7A) corroborated that MIA-induced OA was characterized by severe ECM depletion (ACAN, COL2A1; Figures 7B,C), elevated catabolism (MMP3, MMP13; Figures 7D,E), and suppressed autophagic flux (Beclin1, LC3-II/I, p62; Figures 7F–H) (all *P* < 0.001). PT intervention yielded potent, dose-dependent therapeutic benefits. While PT-L (10 mg/kg) showed only modest restorative effects (*P* < 0.01 for ACAN), PT-M (20 mg/kg) and PT-H (40 mg/kg) significantly reversed these molecular alterations across all categories. Notably, PT-H achieved near-maximal efficacy comparable to RAPA, effectively normalizing both matrix homeostasis and autophagy markers (all *P* < 0.001 vs. MIA). These data confirm that PT alleviates osteoarthritic lesions through dose-dependent autophagy activation, with substantial efficacy emerging at 20 mg/kg and peaking at 40 mg/kg.

Through an integrative approach combining network pharmacology, *in vitro*, and *in vivo* investigations, this study systematically elucidated the chondroprotective mechanism of PT. Our results demonstrate that PT engages and stabilizes p53, promoting its nuclear accumulation to activate the AMPK/mTOR axis, which subsequently enhances autophagy and inhibits chondrocyte apoptosis and ECM degradation. *In vivo* evidence confirms that PT dose-dependently ameliorates OA pathology, with 20–40 mg/kg identified as the optimal therapeutic window. These findings establish PT as a promising, dose-responsive disease-modifying agent for OA management, providing a robust foundation for its clinical translation.

## 4 Discussion

This study provides comprehensive evidence that PT confers chondroprotective effects in OA by activating p53-dependent autophagy. Through an integrated strategy encompassing network analysis, *in vitro* cellular assays, and *in vivo* animal validation, PT was systematically demonstrated to enhance protective autophagic activity via the p53/AMPK/mTOR signaling axis, thereby mitigating cartilage degradation. These findings position PT as a promising disease-modifying therapeutic candidate with clear dose-dependent efficacy, and they offer novel mechanistic insights into autophagy-targeted interventions for OA.

Network analysis identified 238 overlapping targets between PT and OA, with enrichment analyses highlighting the significant involvement of p53 signaling and autophagy-related pathways, providing computational evidence for PT’s multi-target therapeutic mechanism. Among the nine hub genes derived from the PPI network, TP53 exhibited the strongest predicted binding affinity with PT (−7.3 kcal/mol). This poly-targeted pharmacological profile exemplifies the systems pharmacology nature of natural metabolites, which contrasts with the single-target paradigm of conventional synthetic drugs (Hu et al., 2023), and may provide more comprehensive protection against the multifactorial pathogenesis of OA. To rigorously validate the *in silico* predictions, the CETSA was employed. The results demonstrated that PT significantly enhanced the thermal stability of p53 compared with the vehicle control. Such selective thermal stabilization provides direct biophysical evidence that PT physically engages p53 in chondrocytes, thereby corroborating the molecular docking findings.

*In vitro* investigations revealed that PT at 20 μM markedly reversed the IL-1β-induced imbalance in ECM metabolism in chondrocytes by upregulating ACAN and COL2A1 while downregulating MMP3 and MMP13. This bidirectional regulation of ECM turnover offers distinct advantages over conventional therapeutic agents that primarily inhibit matrix degradation (Yang et al., 2023), thereby providing more comprehensive protection for preserving cartilage homeostasis. Importantly, PT’s chondroprotective effects are predominantly mediated through autophagy activation, as evidenced by the upregulation of Beclin1 and the LC3II/I ratio, accompanied by a decrease in p62 accumulation. The partial abrogation of these effects by the autophagy inhibitor 3 MA further substantiates the central role of autophagy in PT-mediated cytoprotection.

A particularly noteworthy and novel observation was the differential regulation of autophagy by IL-1β and PT, despite both inducing p53 expression and nuclear accumulation. Our results demonstrate that although IL-1β enhances p53 nuclear localization, it concurrently suppresses autophagic flux, likely through promoting oxidative stress and activating pro-inflammatory signaling cascades (Guadagno et al., 2015; Rasmussen et al., 2008). Such a pro-oxidative microenvironment may directly compromise the autophagic machinery (Qi et al., 2024). In contrast, PT not only augments p53 nuclear translocation but also selectively enhances its transcriptional activity toward autophagy-related genes (Chen et al., 2017). This mechanistic distinction was confirmed using the p53 transcriptional inhibitor PFT-α, which markedly diminished PT-induced autophagy, thereby establishing p53-dependent transcriptional activation as a pivotal upstream regulator of PT-driven autophagic processes.

Based on experimental evidence and previous research, several plausible mechanisms can be proposed to explain how PT enhances the pro-autophagic function of p53. First, PT appears to facilitate p53 nuclear accumulation, an essential prerequisite for its transcriptional activity. This nuclear retention may be mediated by post-translational modifications, particularly acetylation, which has been reported to prevent p53 nuclear export (Kawaguchi et al., 2006). Second, such acetylation events may further augment the transcriptional capacity of p53, thereby upregulating autophagy-related genes such as SESTRIN2 (Kenzelmann Broz et al., 2013; Haidurov et al., 2025), which subsequently modulate the AMPK/mTOR signaling axis (Li et al., 2021). In addition, the antioxidant properties of PT may contribute to the reduction of excessive ROS, which otherwise interfere with autophagic processes (Xue et al., 2017). While these mechanisms are supported by available data and literature, further investigations focusing on p53 acetylation status and direct promoter-binding interactions would provide more definitive mechanistic validation.

*In vivo* analyses further confirmed the therapeutic efficacy of PT, revealing dose-dependent improvements in joint morphology and histological architecture in the MIA-induced OA model. Notably, significant therapeutic effects were observed at 20 mg/kg, whereas 40 mg/kg produced optimal outcomes, reducing OARSI scores from 16.0 ± 1.7 in the model group to 7.7 ± 1.2. This clear dose–response relationship provides a valuable reference for potential clinical translation. Consistent with *in vitro* observations, PT treatment enhanced autophagic activity in cartilage tissue, as indicated by increased Beclin1 and LC3II/I ratios, accompanied by reduced p62 expression. Unlike synthetic autophagy modulators with narrow therapeutic windows, PT displays a natural multitarget pharmacological profile, offering broader and more balanced chondroprotection against the multifactorial pathogenesis of OA (Yessenkyzy et al., 2020). Although polyphenolic metabolites may exhibit pan-assay interference compound (PAINS) behavior, the CETSA data substantially reduce the likelihood that the observed effects arise from nonspecific assay interference. The observation that PT selectively stabilized p53 without affecting the thermal profile of the abundant housekeeping protein GAPDH confirms that the chondroprotective effects stem from specific drug–target interactions rather than non-specific assay interference.

From a translational perspective, PT demonstrates several advantageous characteristics: (1) as a naturally occurring metabolite, it possesses a favorable safety profile and superior bioavailability compared with resveratrol, supporting its suitability for long-term therapeutic use; (2) the well-defined dose-dependent efficacy within the 20–40 mg/kg range provides a rational foundation for clinical trial design and dose optimization; (3) by modulating upstream molecular mechanisms rather than merely suppressing inflammatory pathways, PT may offer more durable and disease-modifying benefits than conventional NSAIDs (Conaghan et al., 2019). The therapeutic value of PT appears to arise from its capacity to restore balanced autophagy rather than indiscriminately enhancing autophagic flux, as both insufficient and excessive autophagy can compromise chondrocyte homeostasis and promote cartilage degeneration (Li et al., 2024). This balanced regulation of autophagy represents a conceptual shift in OA therapy—from transient symptomatic relief toward targeting the fundamental cellular quality-control mechanisms that preserve cartilage integrity and joint function.

**FIGURE 8:**
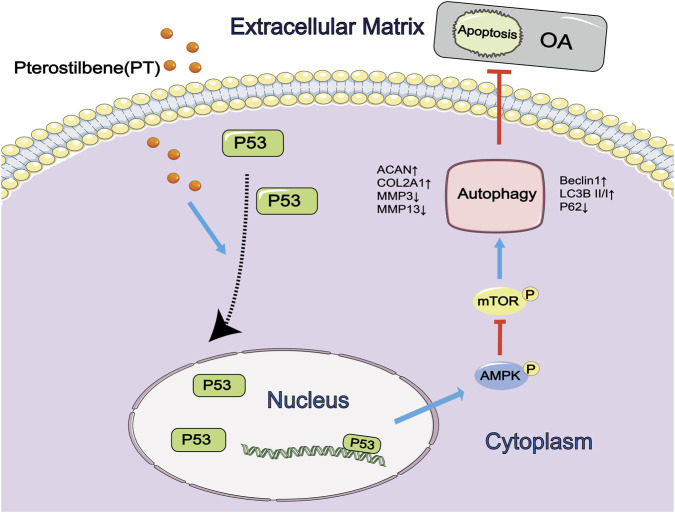
Proposed mechanism by which PT alleviates OA through p53-dependent autophagy activation. Schematic summary illustrating that PT promotes p53 nuclear accumulation and transcriptional activation, leading to AMPK activation and mTOR inhibition, thereby enhancing autophagy and preventing ECM degradation and chondrocyte apoptosis.

The mechanistic insights derived from this study not only reaffirm the traditional use of Pterocarpus santalinus in the management of joint disorders but also provide a scientific foundation for developing evidence-based therapeutic strategies that integrate ethnopharmacological principles with modern precision medicine. A schematic illustration of the proposed mechanism through which PT alleviates OA via p53-dependent autophagy activation is shown in Figure 8, summarizing the principal molecular events elucidated in this research.

Several limitations should be considered when interpreting these findings: (1) although CETSA confirmed the physical interaction between PT and p53, the precise binding kinetics and crystallographic structure of the complex remain to be fully characterized; (2) the study primarily utilized MIA-induced early-stage OA models, which may not fully replicate the biomechanical and pathological complexity of human OA (Aüllo-Rasser et al., 2020); (3) X-ray and micro-CT analyses of subchondral bone were not conducted, limiting evaluation of bone structural changes; (4) the 8-week treatment duration restricted assessment of long-term efficacy and safety; and (5) while the investigation focused on the p53/AMPK/mTOR signaling axis, other potential pathways contributing to the chondroprotective effects of PT require further elucidation.

Future research should aim to address these limitations and broaden understanding of PT’s therapeutic potential: (1) validation of PT’s efficacy in surgically induced OA models that more accurately simulate the mechanical environment of human disease; (2) development of targeted delivery systems, such as nanoparticle-based or hydrogel formulations, to enhance PT bioavailability within avascular cartilage tissue; (3) investigation of PT’s pharmacological effects across different OA stages and in combination with existing therapeutic agents; (4) implementation of comprehensive safety assessments in larger animal models to facilitate clinical translation; and (5) exploration of PT’s actions on other joint compartments, including subchondral bone and synovium, to achieve a more integrated understanding of its multi-tissue therapeutic scope.

## 5 Conclusion

This study elucidates the molecular mechanism through which PT protects chondrocytes from degeneration and apoptosis by promoting p53 nuclear accumulation and transcriptional activation, thereby enhancing autophagy via the AMPK/mTOR signaling axis. The confirmation of specific p53 target engagement complements the dose-dependent chondroprotective efficacy observed *in vivo*, collectively establishing PT as a promising candidate for a disease-modifying osteoarthritis drug. In summary, these findings provide a solid molecular rationale for targeting autophagy in OA therapy and underscore the scientific value of this natural metabolite for clinical translation.

## Data Availability

The original contributions presented in the study are included in the article/Supplementary Material, further inquiries can be directed to the corresponding authors.

## Glossary

3MA: 3-methyladenine
ACAN: aggrecan
BP: biological processes
CC: cellular components
COL2A1: collagen type II alpha 1 chain
CETSA: cellular thermal shift assay
CST: Cell Signaling Technology
CTD: Comparative Toxicogenomics Database
ECM: extracellular matrix
GO: Gene Ontology
H&E: hematoxylin-eosin
IHC: Immunohistochemistry
IL-1β: interleukin-1β
KEGG: Kyoto Encyclopedia of Genes and Genomes
MF: molecular functions
MIA: monosodium iodoacetate
MMP3: matrix metalloproteinase 3
MMP13: matrix metalloproteinase 13
NSAIDs: non-steroidal anti-inflammatory drugs
OA: osteoarthritis
OARSI: Osteoarthritis Research Society International
OD: optical density
OMIM: Online Mendelian Inheritance in Man
PAINS: pan-assay interference compound
PFT-α: pifithrin-α
PPI: protein-protein interaction
PT: pterostilbene
PT-H: high-dose PT group
PT-L: low-dose PT group
PT-M: medium-dose PT group
RAPA: rapamycin
ROS: reactive oxygen species
SEM: standard error of the mean
TTD: Therapeutic Target Database
WB: Western blotting
