# Supplementary figures and images for: Pterostilbene attenuates osteoarthritis progression through p53-dependent autophagy activation: evidence from network analysis and experimental validation
Source: Front Pharmacol. 2026 Jan 23;17:1686555. doi: 10.3389/fphar.2026.1686555 (PMC12876155; doi:10.3389/fphar.2026.1686555)

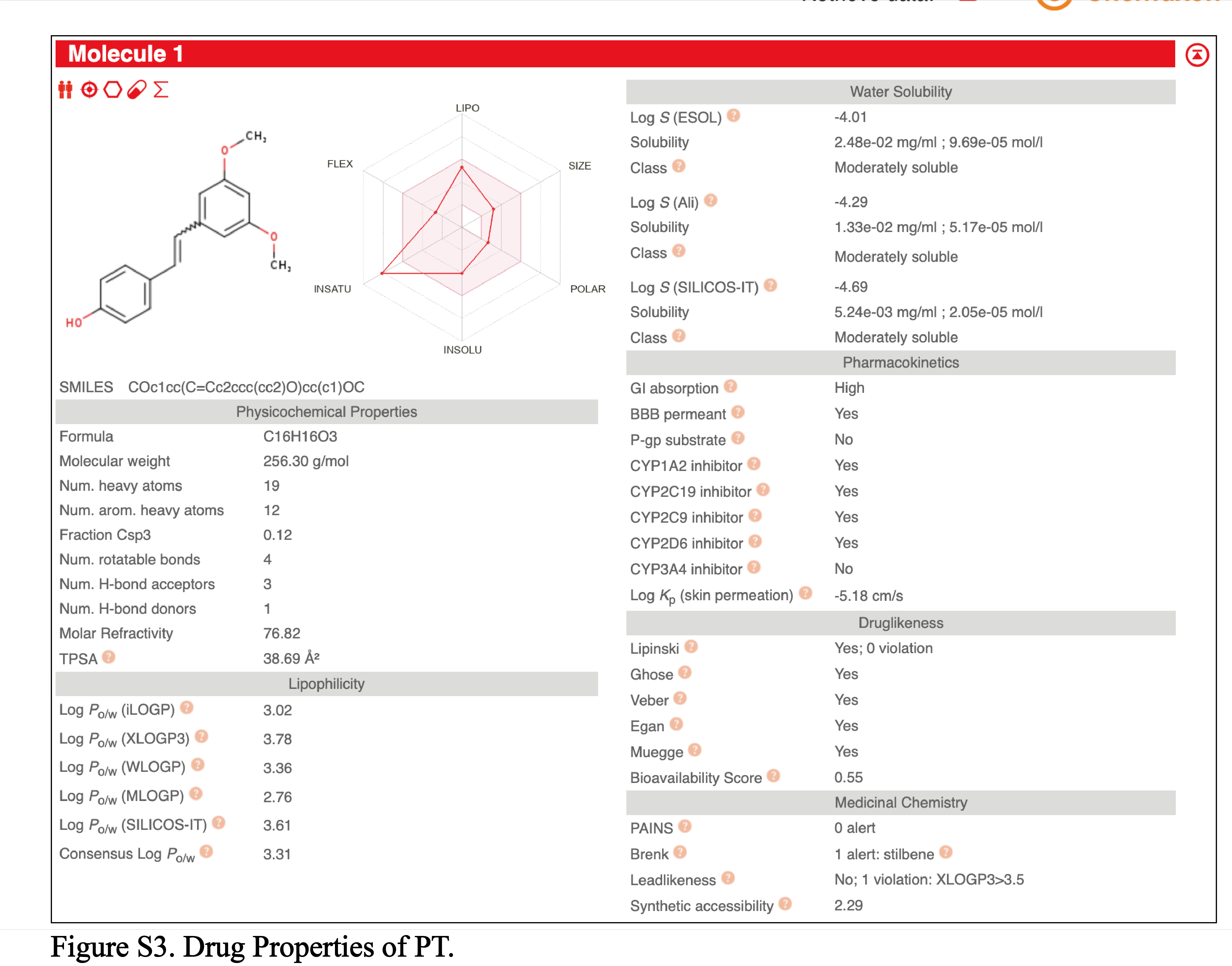

Supplement: Supplementary file 1 [file Image3.tiff]

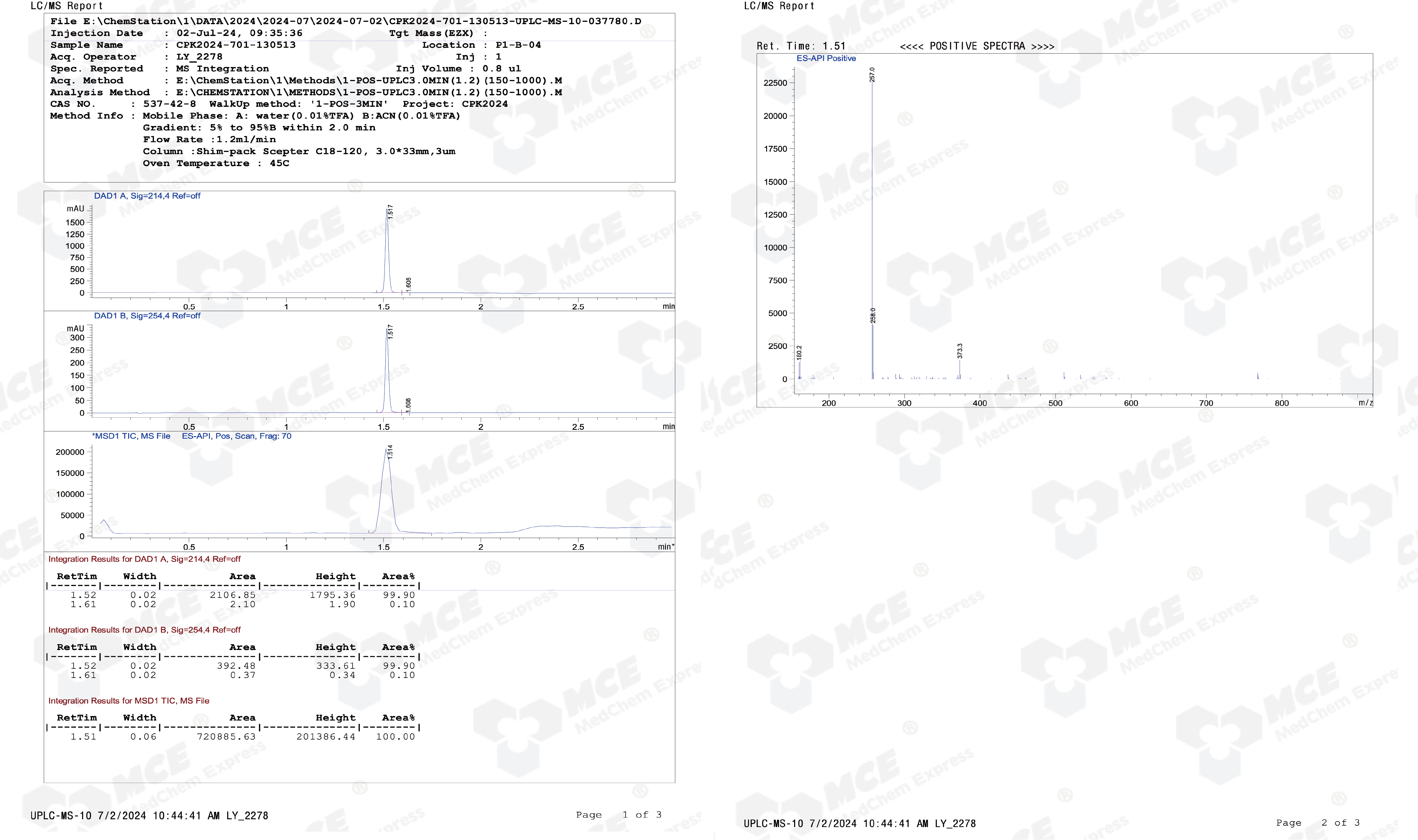

Supplement: Supplementary file 2 [file Image1.tiff]

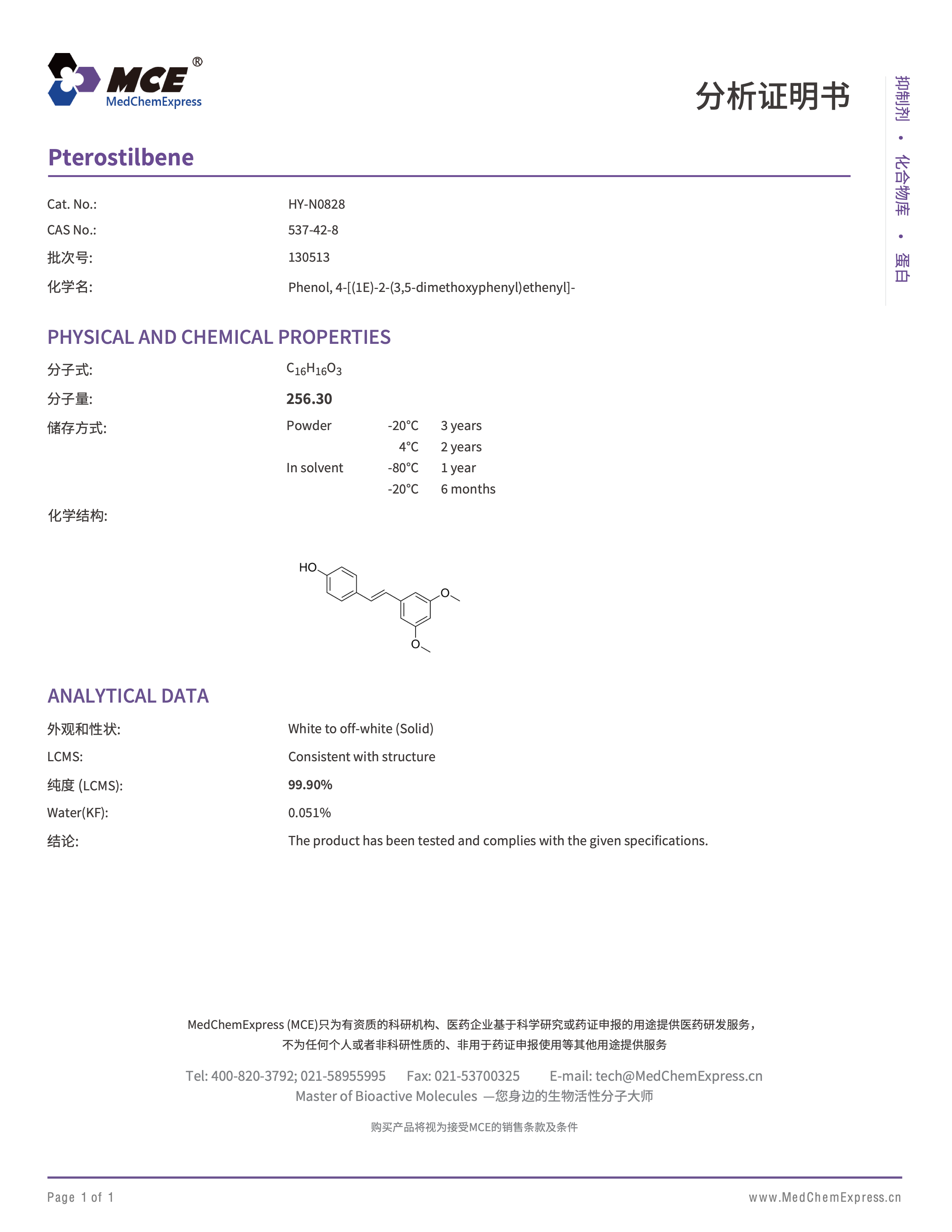

Supplement: Supplementary file 4 [file Image2.tiff]

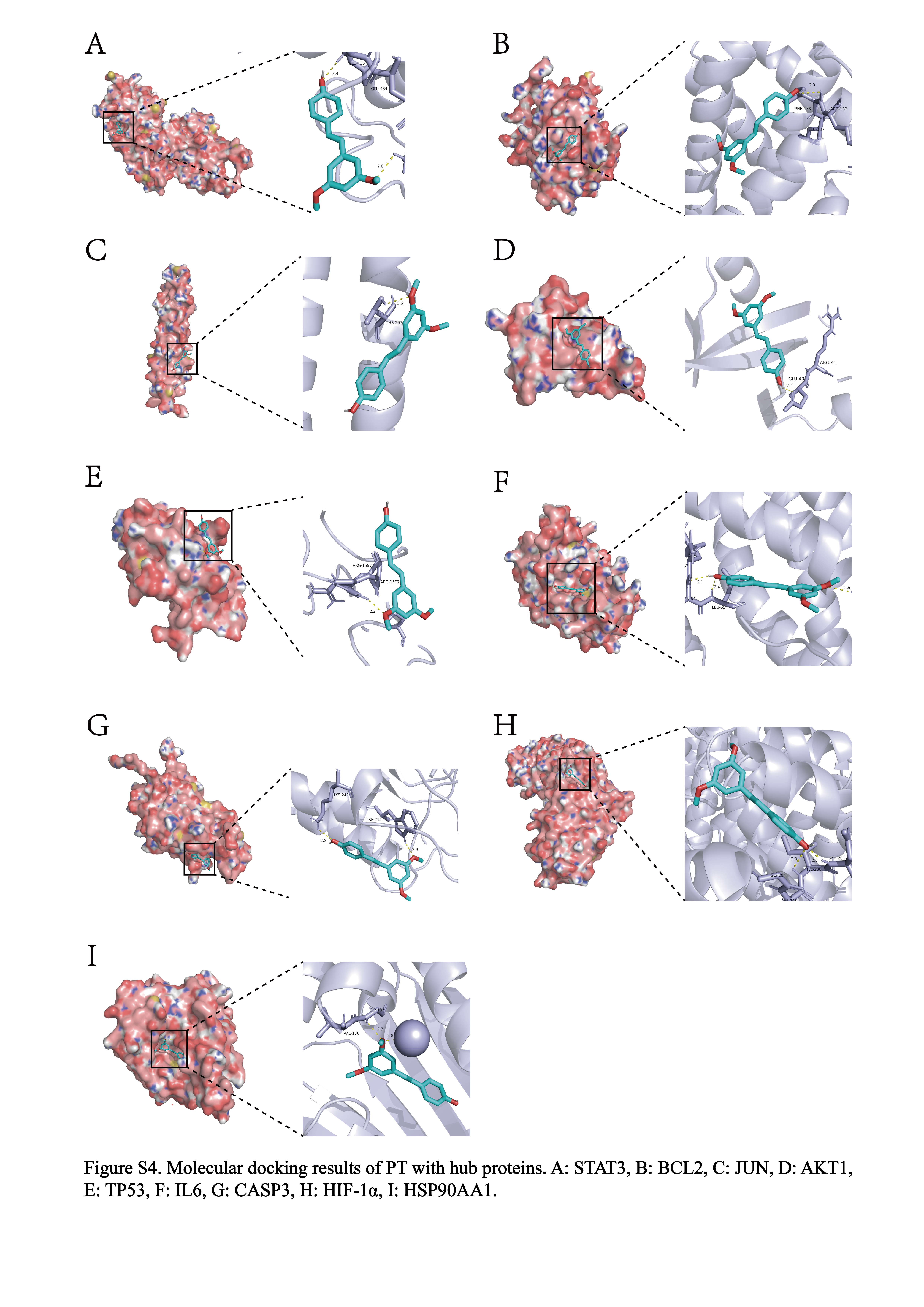

Supplement: Supplementary file 5 [file Image4.tiff]
