# Supplementary material for: Pterostilbene attenuates osteoarthritis progression through p53-dependent autophagy activation: evidence from network analysis and experimental validation
Source: Front Pharmacol. 2026 Jan 23;17:1686555. doi: 10.3389/fphar.2026.1686555 (PMC12876155; doi:10.3389/fphar.2026.1686555)
Supplement: Supplementary file 3 [file Table1.docx]

Table S1. Autophagy-related scores of the 9 hub proteins evaluated by VarElect.

| Hub protein | PDB IDs | Description | -LOG10(p) | Score |
| --- | --- | --- | --- | --- |
| STAT3 | 6NJS | Signal Transducer And Activator Of Transcription 3 | 2.53 | 7.74 |
| BCL2 | 1G5M | BCL2 Apoptosis Regulator | 2.97 | 17.61 |
| JUN | **1JUN** | Jun Proto-Oncogene, AP-1 Transcription Factor Subunit | 1.91 | 3.49 |
| AKT1 | 1H10 | AKT Serine/Threonine Kinase 1 | 2.68 | 10.13 |
| TP53 | 2G3R | Tumor Protein P53 | 2.80 | 12.47 |
| IL6 | 1IL6 | Interleukin 6 | 2.26 | 5.30 |
| CASP3 | 1GFW | Caspase 3 | 2.41 | 6.47 |
| HIF1A | 8HE0 | Hypoxia Inducible Factor 1 Subunit Alpha | 2.45 | 6.83 |
| HSP90AA1 | 2CG9 | Heat Shock Protein 90 Alpha Family Class A Member 1 | 2.51 | 7.48 |

Table S2. Binding energy of hub proteins.

| Hub protein | STAT3 | BCL2 | JUN | AKT1 | TP53 | IL6 | CASP3 | HIF1A | HSP90AA1 |
| --- | --- | --- | --- | --- | --- | --- | --- | --- | --- |
| Binding energy （kcal/mol） | -5.0 | -6.3 | -5.0 | -5.7 | -7.3 | -6.1 | -6.6 | -5.2 | -6.9 |
